# Systematic Analysis of tRNA-Derived Small RNAs Reveals the Effects of Xuefu-Zhuyu Decoction on the Hippocampi of Rats after Traumatic Brain Injury

**DOI:** 10.1155/2022/5748719

**Published:** 2022-09-17

**Authors:** Feng Dai, Tao Tang, Ruohuang Lu, Pengfei Li, Dandan Feng, Mingrui Hu, Yang Wang, Pingping Gan

**Affiliations:** ^1^Institute of Integrative Medicine, Department of Integrated Traditional Chinese and Western Medicine, Xiangya Hospital, Central South University, 410008 Changsha, China; ^2^Department of Emergency, Xiangya Hospital of Central South University, 410008 Changsha, China; ^3^Department of Stomatology, The Third Xiangya Hospital, Central South University, 410013 Changsha, China; ^4^Department of Respiratory and Critical Care Medicine, The First Affiliated Hospital of Zhengzhou University, 450052 Zhengzhou, China; ^5^Department of Oncology, Xiangya Hospital, Central South University, 410008 Changsha, China

## Abstract

**Background:**

Traumatic brain injury (TBI) is one of the most common neurosurgical diseases and refers to brain function impairment or brain pathological changes induced by external causes. A traditional Chinese medicine, Xuefu-Zhuyu Decoction (XFZYD), has been indicated to harbor therapeutic properties against TBI. Transfer RNA (tRNA)-derived small RNAs, that is, tsRNAs (a group of small RNAs derived from tRNAs), are multifunctional regulatory noncoding RNAs generated under pressure and implicated in the progression of TBI.

**Methods:**

A TBI model was successfully constructed using rats. We further performed sequencing and omics analyses to identify novel tsRNAs as drug targets for XFZYD therapy against TBI in the rat hippocampus. qPCR assays were used to further verify the experimental results. Gene Ontology (GO) was used to analyze the signaling pathways of downstream target genes of tsRNAs in the XFZYD-regulated TBI model. qPCR was used to detect the influence of overexpressed tsRNA mimics/inhibitors on their target genes in PC12 cells.

**Results:**

Our RNA-Seq data illustrate that 11 tsRNAs were mediated by XFZYD. The experimental data revealed AS-tDR-002004 and AS-tDR-002583 as potential targets for XFZYD therapy and showed that they influenced TBI via the cadherin signaling pathway, cocaine addiction, circadian entrainment, and the nicotine pharmacodynamics pathway. We also confirmed that Pi4kb, Mlh3, Pcdh9, and Ppp1cb were target genes of 2 XFZYD-regulated tsRNAs in the hippocampus of a rat model and PC12 cells. Furthermore, biological function analysis revealed the potential therapeutic effects of tsRNAs, and the results showed that Mapk1 and Gnai1 were related genes for XFZYD therapy against TBI.

**Conclusion:**

Our work successfully illuminates the efficiency of XFZYD in the treatment of TBI. The experimental data revealed AS-tDR-002004 and AS-tDR-002583 as potential targets for XFZYD therapy and showed that they influenced TBI via the cadherin signaling pathway, cocaine addiction, circadian entrainment, and the nicotine pharmacodynamics pathway in a TBI rat model.

## 1. Introduction

Traumatic brain injury (TBI) is a global public health problem characterized by brain function impairment or brain pathological changes induced by external causes [1]. As one of the most common neurological diseases, TBI can lead to temporary or even permanent nerve dysfunction in the brain [2]. In addition to its acute onset and rapid progression, TBI also features high mortality and disability rates. Globally, TBI results in approximately 10 million hospitalizations and/or deaths every year, with an estimated 57 million people still suffering from the consequences of the condition [3]. Unfortunately, it remains challenging to clinically improve the survival rate of patients with TBI and to promote the recovery of nerve function. However, the effects of currently available treatment modalities on the recovery of nerve function are limited [4, 5]. TBI is a diverse group of sterile injuries caused by primary and secondary mechanisms that contribute to cell death, inflammation, and neurologic dysfunction in patients of all demographics [5, 6]. The primary injury of TBI has been attributed to mechanical stress or shear force, yet therapeutic drugs are not available at present. The secondary injury of TBI involves multiple processes, and the current therapeutic strategies include activation of inflammatory and immune responses, calcium overload, glutamate toxicity, and mitochondrial dysfunction [5, 7, 8]. Drugs that combat secondary injury due to TBI include free-radical scavengers, antagonists of N-methyl-D-aspartate, and calcium channel blockers [5, 9–11]. Nevertheless, the outcomes are not effective. Therefore, it is urgent to develop novel therapeutic strategies and drugs for the management of TBI. The traditional Chinese medicine Xuefu-Zhuyu Decoction (XFZYD) can invigorate the circulation of blood without any consumption of blood, dissipate blood stasis, and promote angiogenesis [5, 12]. Oral administration leads to peripheral vasodilation and enhanced aerobic catabolism, alleviating tissue edema, exudation, and inflammatory reactions, by which clinical symptoms can be improved [5, 13, 14]. Recent research has demonstrated the treatment effects of XFZYD on TBI, yet the underlying mechanism remains unknown [15]. Transfer RNA (tRNA)-derived small RNAs (tsRNAs), also known as tRNA-derived fragments (tRFs), are a group of small RNAs derived from tRNAs that are multifunctional regulatory inhomogeneous small noncoding RNAs (ncRNAs) with a length of 18–40 nucleotides [16, 17]. They are primarily considered a byproduct of randomly cleaved tRNAs. According to their length and cleavage site, tsRNAs can usually be grouped into tRFs and tRNA halves (tiRNAs), most of which are generated under pressure [16, 17]. TsRNAs harbor potential regulatory functions implicated in diverse biological and pathological processes. Recent investigations have emerged regarding new functions of tsRNAs based on tsRNA sequences, RNA modification, and structure [5, 18, 19]. The involvement of tsRNAs in tumorigenesis has been indicated in different contexts, including chronic lymphocytic leukemia, lung cancer, breast cancer, and ovarian cancer [5, 20, 21]. Moreover, tsRNAs have been recognized to play important roles in spermatogenesis, differentiation, and metabolic processes [22]. Additionally, omics has provided a technical means for the investigation of tsRNAs in nerves and brains [5, 23]. Notably, some tsRNAs have been revealed to participate in the mediation of neurological disorders and pathological processes of traumatic spinal cord injury. We followed the methods of Pengfei Li et al. [24]. They proposed another traditional Chinese medicine, Buyang-Huanwu Decoction, as a new therapeutic target for cerebral hemorrhage based on a systematic analysis of tsRNAs [24, 25]. We endeavored to identify target tsRNAs of the traditional Chinese medicine XFZYD for the treatment of TBI and to unravel the underlying mechanism, offering a promising candidate for the management of TBI.

## 2. Methods

### 2.1. Preparation of XFZYD

The matching prescription of XFZYD was as follows: peach seeds, 12 g; safflower, Angelica sinensis, and Rehmannia roots, 9 g; Ligusticum chuanxiong and Platycodon, 4.5 g; red peony roots, fructus aurantii, and licorice roots, 6 g; and bupleurum roots, 3 g. The plant seeds and tissue were obtained commercially from the pharmacy of Xiangya Hospital, Central South University. The plant material used in this study was on sale in the pharmacy of Xiangya Hospital, which is according to the 10th Pharmacopoeia Commission of the People's Republic of China.

### 2.2. Traumatic Brain Injury (TBI)

All animal protocols were approved by the Committee on the Use and Care of Animals of CSU and conformed to the Guide for the Care and Use of Laboratory Animals of the National Institutes of Health (NIH Publication No. 85–23, revised 1996). The protocol was approved by the Medical Ethics Committee of Xiangya Hospital of Central South University. Adult male Sprague–Dawley rats (180–220 g) were obtained from Hunan SJA Laboratory Animal Co., Ltd. (SCXK (XIANG) 2019–0004) and housed in SPF conditions in the Laboratory Animal Centre of Central South University (CSU, SYXK (XIANG) 2015–0017). Rats were randomly divided into three groups: sham, CCI (controlled cortical impact), and XFZYD groups, with 4 rats per group. CCI was performed as described previously under 3% pentobarbital sodium (60 mg/kg) anesthesia [24]. The parameters were as follows: impact depth, 5.0 mm; striking speed, 6.0 m/sec; dwell time, 50 msec. The sham-operated rats were subjected to the same anesthesia and craniotomy except for cortical impact. In the XFZYD group, rats were intragastrically given 1.52 g/kg (equivalent to 9 g/kg of raw herbs) XFZYD. The rats in the sham and CCI groups were treated with equal volumes of distilled water. The mNSS (modified neurological severity score) was used to assess the movement, sensation, and reflexes of TBI rats.

### 2.3. tsRNA Sequencing (tsRNA-Seq)

**Figure 1:**
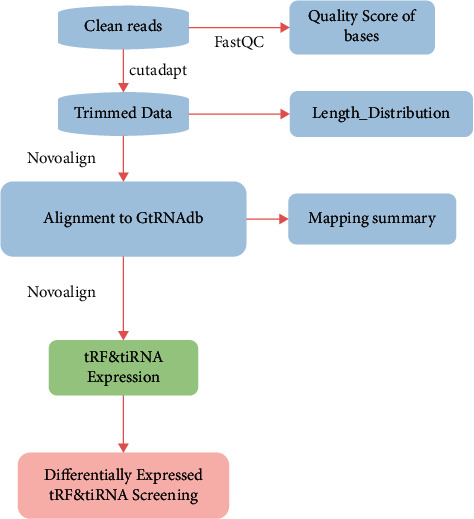
Study design schematic. TBI, traumatic brain injury; XFZYD, Xuefu-Zhuyu Decoction; FC, fold change; qPCR, quantitative real-time PCR.

Rats were deeply anesthetized and sacrificed by intraperitoneal injection of pentobarbital and perfused with ice-cold saline. Then, hippocampal tissues surrounding the hemorrhagic region were harvested for subsequent detection. Total RNA was extracted from the sham, TBI, and XFZYD groups (*n* = 4 each group) according to the manufacturer's instructions (Qiagen, USA). Subsequently, using rtStar tRF & tiRNA Pretreatment Kit protocols (Arraystar, USA), some RNA modifications that might interfere with small RNA-sequencing library construction were removed (Figure 1). cDNA was then synthesized and amplified using Illumina's proprietary reverse transcription primers and amplification primers. Afterward, 135–170 nt PCR-amplified fragments (corresponding to 15–50 nt small RNAs) were extracted and purified from the PAGE gel using an automated gel cutter. The libraries were qualified and absolutely quantified using an Agilent BioAnalyzer 2100. Finally, the sequencing run was performed on an Illumina NextSeq 500 system using a NextSeq 500/550 V2 kit (Illumina, USA). Sequencing was carried out by running 50 cycles. RNA-seq data have been submitted to NCBI with the accession number SUB8928870.

### 2.4. Data Analysis

The raw sequencing data that passed the Illumina chastity filter were used for the following analysis. After Illumina quality control, the sequencing reads were 5′, 3′-adaptor trimmed and filtered for over 15 nt by Cutadapt software. Then, using NovoAlign software (v2.07.11), trimmed reads were aligned to mature-tRNA and pre-tRNA sequences from GtRNAdb (http://gtrnadb.ucsc.edu/). The exactly matched reads were thought to be tsRNAs. Additionally, we employed statistical analysis to annotate tRFSs properly (Supplementary File 1). Moreover, tsRNA abundance levels could be measured and normalized as tag counts per million total aligned tRNA reads (TPM). tsRNA abundance profiling and differential expression analysis were calculated by the average TPM. Fold changes (FC; TBI versus sham or XFZYD versus TBI) were used to compare two groups of profile differences. In addition, FC > 1.3 and *p* value < 0.05 were considered significantly differentially expressed, and these tsRNAs were chosen for further analysis. In the standard of FC > 1.3 and *p* < 0.05, we identified TBI-induced tsRNAs (TBI versus sham) and XFZYD-induced tsRNAs (XFZYD versus TBI).

### 2.5. Target Prediction of Treatment-Related tsRNAs

We used two common algorithms to predict tsRNA targets, TargetScan (http://www.targetscan.org) and miRanda (http://www.microrna.org). Additionally, to reduce false-positive results, only genes predicted by both software programs were considered targets of tsRNAs. The network illustration was visualized with Cytoscape software (version 3.5.1, the Cytoscape Consortium, San Diego, CA, USA).

### 2.6. Gene Ontology (GO) and Pathway Enrichment Analysis

The functional enrichment tool DAVID (DAVID, https://david.ncifcrf.gov/, ver. 6.8) was used to calculate both the KEGG pathway and GO biological processes (BP) enrichments.

### 2.7. Quantitative Real-Time Polymerase Chain Reaction (qRT-PCR)

Total RNA was extracted using TRIzol extraction reagent (Invitrogen, Grand Island, NY, USA) according to the manufacturer's instructions. RNA purity and concentration were photometrically tested. RNA was reverse-transcribed into cDNA using a RevertAid First Strand cDNA Synthesis Kit (Takara, Japan). qPCR was performed using SYBR Green qPCR Supermix (Takara, Japan) with an ABI 7500 RT–PCR machine (Bio-Rad, CA, USA). The gene expression levels were calculated relative to *β*-actin using the 2^−ΔΔCq^ method. The cycling conditions were as follows: incubation at 95°C for 10 min, followed by 40 cycles of 95°C for 10 s, 60°C for 60 s, and 95°C for 15 s. The primer sequences were as follows:

*β*-actin F: 5' ACATCCGTAAAGACCTCTATGCC 3', R: 5' TACTCCTGCTTGCTGATCCAC 3'; Pdyn F: 5' CACGGAACTGACCAAGCTCT 3', R: 5' GTCAGTGCCCAGTAGCTCAG 3'; Mapk1 F: 5' TGAAGACACAGCACCTCAGCAATG 3', R: 5' GGTGTTCAGCAGGAGGTTGGAAG 3'; Pcdh9 F: 5'GTGCTTGGTTTTGGGTCACT 3', R: 5' CGGTCATTGAACTGGTTCCT 3'; Gnai1 F: 5' GTGCTTGGAGCCCGCACTCGG 3', R: 5' AGATTCACCAGCACCGAGCAGCA 3'; Pi4bk F: 5'GCCCACCAGGGAATAA3′', R: 5' TCCACTACTGTATCTCCCAT3'; Mhl3 F: 5' GACGTATGTTCCCGATTTTGTCA 3', R: 5' GCTTCAGAGCTGATATAGCCACT 3'; Ppp1cb F: 5' TGGACAGCCTCATCAC 3', R: 5' TTCAGCTCCCCGTCCGCCAT 3'.

### 2.8. Cell Culture

HyClone™ Dulbecco's modified Eagle's medium (DMEM) (GE Healthcare Life Science) with 10% fetal bovine serum (FBS) (Corning) was used for PC12 cell culture. The PC12 cell line was purchased from American Type Culture Collection (ATCC, USA). Cells were maintained at 37°C in a humidified incubator with 5% CO2, and cell passage was performed when the cell density was ∼90%.

### 2.9. Statistical Analysis

All results are expressed as the mean ± SE. Statistical analysis of the data was performed using GraphPad Prism 7 (Sorrento Valley, CA, USA). Comparisons between samples were performed by one-way ANOVA with Tukey's multiple comparison tests. Differences were considered significant at *p* < 0.05.

## 3. Results

### 3.1. Effects of XFZYD on Neurological Recovery after TBI

**Figure 2:**
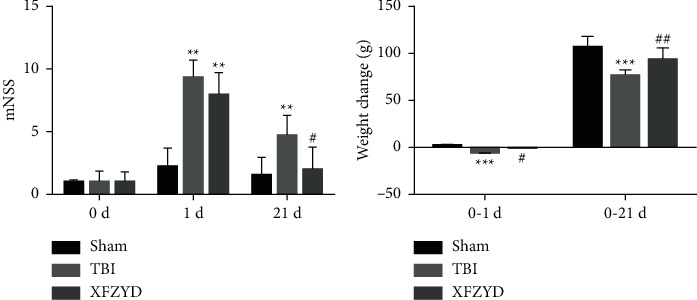
Effects of XFZYD on neurological recovery after TBI. On day 1, the mNSS (a) and weight change (b) of the TBI and XFZYD groups were significantly different from those of the sham group, which indicated successful establishment of the TBI model. On day 21, the mNSS (a) and weight change (b) of the sham group and XFZYD group showed significantly different values than those of the TBI group, which suggested the therapeutic effects of XFZYD for functional recovery after TBI. Data are presented as the mean ± SEM (*n* = 15 each group); ^*∗*^*p* < 0.05, ^*∗∗*^*p* < 0.01, and ^*∗∗∗*^*p* < 0.001 indicate significant differences compared with the sham group; ^#^*p* < 0.05 indicates a significant difference compared with the TBI group, mNSS, modified neurological severity score; 0 d, the day of surgery but before anesthesia; 1 d and 21 d, the 1^st^ and 21^st^ days after TBI; weight change, the difference between weight values at 1 and 21 days and 0 days.

The laboratory rats were divided into 3 groups: the sham group, the TBI group, and the XFZYD group (*n* = 4 each group). The degree of nerve injury was assessed by the modified neurological severity score (mNSS) and weight changes. On Day 0, mNSS scores were calculated to show the baseline levels of normal rats, and there was no statistically significant difference between the three groups (*p* > 0.05) (Figure 2(a)). Compared with the sham group, the mNSS scores on the 1^st^ day were significantly higher in the TBI group, while weight change was markedly decreased in the TBI group (*p* < 0.001) (Figure 2(b)), indicating the successful establishment of a rat model of TBI. To determine whether long-term XFZYD therapy was beneficial to TBI in rats, XFZYD therapy was applied to rats with TBI for 1–21 days. On the 21^st^ day, the mNSS scores of TBI rats were still higher than those of sham rats (*p* < 0.01) (Figure 2(a)). However, after 21 days of treatment with XFZYD, the scores of XFZYD rats significantly declined in mNSS (*p* < 0.05). Additionally, TBI worsened weight gain compared with sham treatment (*p* < 0.001). In addition, XFZYD treatment improved the increase in TBI (*p* < 0.05) (Figure 2(b)). Taken together, these findings suggested that long-term XFZYD therapy significantly alleviated rat behaviors during recovery when compared with the TBI group. Therefore, the following experiments were performed using 21-day therapy with XFZYD.

### 3.2. XFZYD Therapy Changed the Expression Profiles of tsRNAs

**Figure 3:**
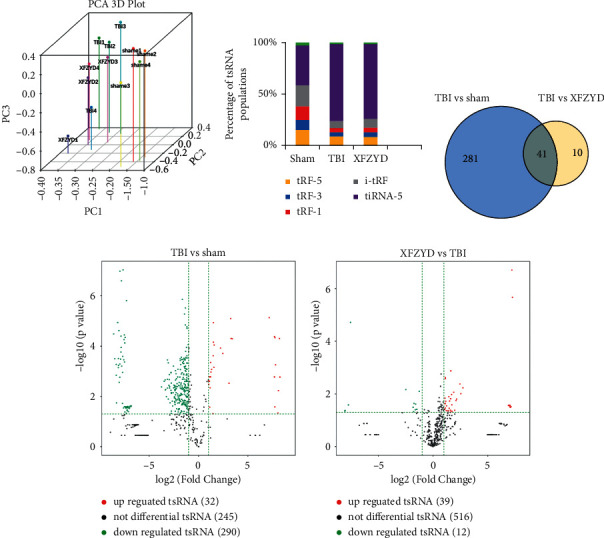
XFZYD therapy changed the expression profiles of tsRNAs. (a) PCA plot illustrating the clustering of the 4 replicates of each group and assessing the variation and reproducibility (FC > 1.3 and *p* < 0.05). The red points represent the sham group; green, the TBI group; blue, the XFZYD group. (b) Histogram showing the abundance of each tsRNA subtype in the 3 groups. XFZYD treatment did not obviously reverse these changes in the TBI group. (c) Venn diagram showing the total number of identified tsRNAs in the brain tissues of the XFZYD groups in comparison to the TBI group. (d) Volcano plot showing the significantly changed tsRNAs between the TBI and sham groups (FC > 1.3 and *p* < 0.05) and the significantly changed tsRNAs between the XFZYD and TBI groups (FC > 1.3 and *p* < 0.05).

To identify targets of XFZYD therapy against TBI, the tsRNA-Seq method was introduced to detect the expression profile changes of tsRNAs in the rat hippocampi of the sham, TBI, and XFZYD groups. The original tsRNA-Seq data have been submitted to the Gene Expression Omnibus. There were 365 precisely matched tsRNAs identified from tsRNA-Seq, 322 for the sham group, 51 for the TBI group, and 197 for the XFZYD group (Figure 3(a) and Supplementary File 2). Generally, tsRNA abundance was significantly increased in the TBI group to various degrees compared with the sham group. Although rats following XFZYD therapy could not match up to rats in the sham group, the changes induced by TBI were slightly reversed (Figure 3(b)). When comparing the TBI group with the sham group, the abundances of 322 tsRNAs were altered. In comparison to the TBI group, the abundances of 51 tsRNAs were changed in the XFZYD group. Hence, 41 tsRNAs were obtained in the intersection that might respond to XFZYD therapy against TBI (Figure 3(c)). Additionally, we identified significantly dysregulated tsRNAs in TBI rats: 5 were upregulated, while 7 were downregulated (TBI versus sham; FC > 1.3 and *p* < 0.05) (Figure 3(d) and Supplementary File 3). After XFZYD treatment, 11 tsRNAs were obviously changed: 10 were upregulated, while 1 was downregulated (XFZYD versus TBI; FC > 1.3 and *p* < 0.05) (Figure 3(d)).

### 3.3. Identification and Confirmation of Related tsRNAs by XFZYD Therapy

**Figure 4:**
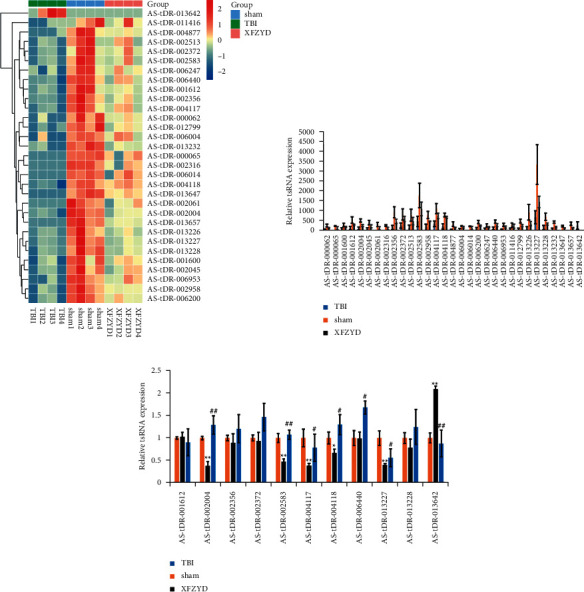
XFZYD treatment-related tsRNAs and qPCR confirmation. The significantly changed tsRNAs are shown in the heatmap (a) and histogram (b). One upregulated tsRNA (AS-tDR-013642) and 30 downregulated tsRNAs (TBI versus sham; fold change >1.5, *p* < 0.05) were selected. (c) Quantitative polymerase chain reaction (qPCR) was conducted to verify the selected 11 tsRNAs. Data are presented as the mean ± SEM (*n* = 3 each group); ^*∗*^*p* < 0.05 and ^*∗∗*^*p* < 0.01 indicate significant differences compared with the sham group; ^0^*p* < 0.05 and ^##^*p* < 0.01 indicate significant differences compared with the TBI group.

Afterward, we aimed to identify the significantly upregulated/downregulated tsRNAs in the TBI group compared with those in the sham group as well as the significantly downregulated/upregulated tsRNAs in the XFZYD group compared with those in the TBI group. Accordingly, 31 tsRNAs were found, as shown by the heatmaps in Figure 4(a). After screening, one upregulated tsRNA (AS-tDR-013642) and 30 downregulated tsRNAs (TBI versus sham; fold change >1.5, *p* < 0.05) were selected (Supplementary File 3). Moreover, 11 tsRNAs were finally obtained with a tag count ≠ 0 as the threshold since sequences from tsRNA-Seq were required to be precisely matched with those of derived tsRNAs, including 10 upregulated tsRNAs (AS-tDR-001612, AS-tDR-002004, AS-tDR-002356, AS-tDR-002372, AS-tDR-002583, AS-tDR-004117, AS-tDR-004118, AS-tDR-006440, AS-tDR-013227, and AS-tDR-013228) and one downregulated tsRNA (AS-tDR-013642) (XFZYD versus TBI; fold change >1.5, *p* < 0.05).

Furthermore, quantitative polymerase chain reaction (qPCR) was conducted to verify the 11 selected tsRNAs (Figure 4(c)). AS-tDR-002004 and AS-tDR-002583 were significantly downregulated in the TBI group compared with the sham group (*p* < 0.05). After XFZYD therapy, AS-tDR-002004 and AS-tDR-002583 were significantly upregulated (*p* < 0.01). Conclusively, these results revealed AS-tDR-002004 and AS-tDR-002583 as potential targets of XFZYD therapy against TBI.

### 3.4. Identification and Confirmation of Related Target Genes of tsRNAs by XFZYD Therapy

**Figure 5:**
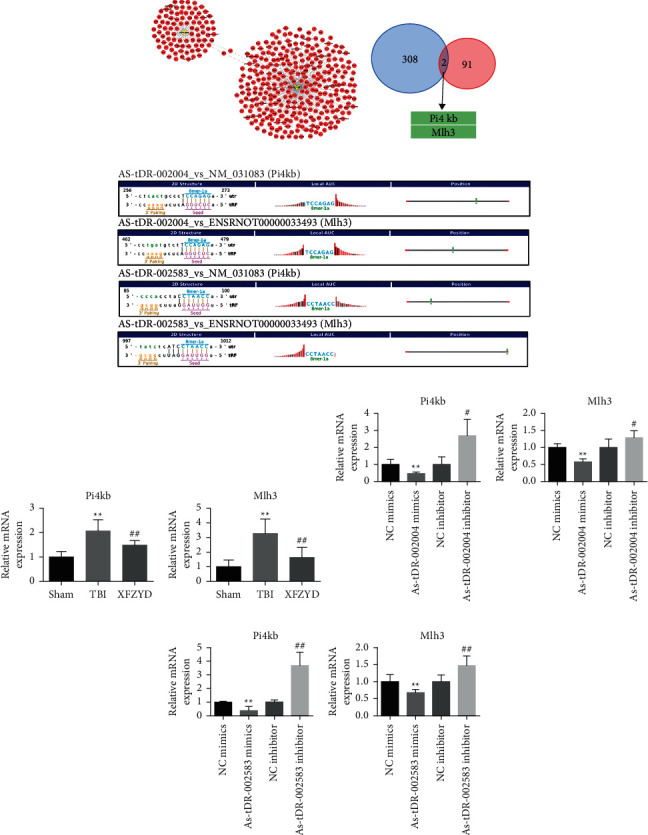
Analysis of target genes of XFZYD therapy-related tsRNAs. (a) Target genes of AS-tDR-002004 and AS-tDR-002583 were analyzed by TargetScan. (b) Venn plot indicating that two common target genes Pi4kb and Mlh3 of AS-tDR-002004 and AS-tDR-002583 were predicted. (c) The binding region and seed sequence are shown. (d) The mRNA expression levels of Pi4kb and Mlh3 in the sham, TBI, and XFZYD groups by qPCR. ^*∗*^*p* < 0.05 and ^*∗∗*^*p* < 0.01 indicate significant differences compared with the sham group; ^#^*p* < 0.05 and ^##^*p* < 0.01 indicate significant differences compared with the TBI group. (e, f) qPCR detected target gene levels of overexpressed or inhibited AS-tDR-002004/AS-tDR-002583 in PC12 cells. ^*∗*^*p* < 0.05 and ^*∗∗*^*p* < 0.01 indicate significant differences compared with NC mimics; ^#^*p* < 0.05 and ^##^*p* < 0.01 indicate significant differences compared with NC inhibitor.

Subsequently, target genes of AS-tDR-002004 and AS-tDR-002583 were analyzed by TargetScan, the results of which revealed 310 and 90 transcripts of AS-tDR-002004 and AS-tDR-002583, respectively (Figure 5(a) and Supplementary File 4). Two common target genes, Pi4kb and Mlh3, were identified (Figure 5(b)). The binding sites are depicted in Figure 5(c). Thereafter, a qPCR assay was performed to verify that, during XFZYD therapy, Pi4kb and Mlh3 may be candidate related target genes of tsRNAs. Our results showed that both Pi4kb and Mlh3 were upregulated in the TBI group and downregulated after XFZYD therapy compared with the sham group (*p* < 0.05) (Figure 5(d)). The resulting tsRNA abundance was changed oppositely (*p* < 0.05). Additionally, tsRNAs were overexpressed or inhibited in the PC12 cell line to explore the abundance alterations of their target genes for further verification (Figures 5(e) and 5(f)). After transfection of the AS-tDR-231002004 mimic or the AS-tDR-002583 mimic, the expression levels of Pi4kb and Mlh3 were diminished (*p* < 0.05), while the delivery of the AS-tDR-002004 inhibitor or the AS-tDR-002583 inhibitor significantly elevated the expression levels of Pi4kb and Mlh3 (*p* < 0.05) (Figures 5(e) and 5(f)). These in vitro results were consistent with the qPCR results in the rat model. The abovementioned findings demonstrated that AS-tDR-002004 and AS-tDR-002583 might be involved in XFZYD therapy against TBI by regulating their common target genes, that is, Pi4kb and Mlh3.

### 3.5. Analysis of Target Genes of XFZYD Therapy-Related tsRNAs

**Figure 6:**
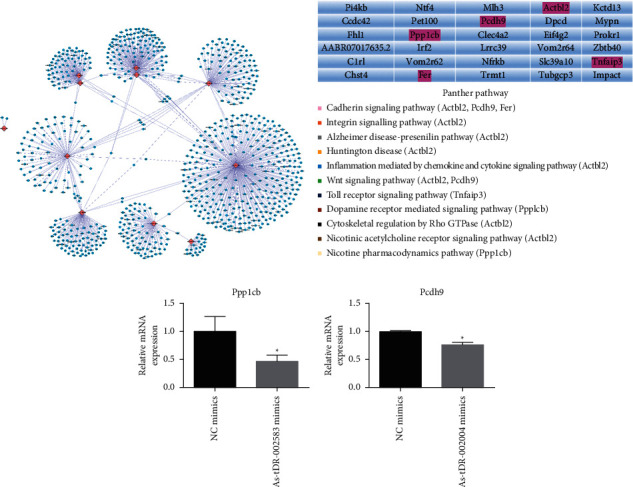
Analysis of target genes of XFZYD therapy-related tsRNAs. (a) The TargetScan and miRanda databases were used to predict 11 XFZYD-related target genes of 11 selected tsRNAs. (b) Gene Ontology (GO) pathway analysis of 30 target genes. (c) qPCR detected target gene levels of overexpressed or inhibited AS-tDR-002004/AS-tDR-002583 in PC12 cells. ^*∗*^*p* < 0.05 and ^*∗∗*^*p* < 0.01 indicate significant differences compared with NC mimics; ^#^*p* < 0.05 and ^##^*p* < 0.01 indicate significant differences compared with NC inhibitor.

Although the detailed regulatory mechanism of tsRNAs remains less studied, a recent surge of evidence has proven the similar functions between tsRNAs and microRNAs considering their transcriptional inhibitory action. However, seed sequences can potentially inhibit the overall translation activity of target messenger RNAs by complementary base pairing. The TargetScan and miRanda databases were used to predict 11 XFZYD-related target genes of 11 selected tsRNAs, including AS-tDR-001612, AS-tDR-002004, AS-tDR-002356, AS-tDR-002372, AS-tDR-002583, AS-tDR-004117, AS-tDR-004118, AS-tDR-006440, AS-tDR-013227, AS-tDR-013228, and AS-tDR-013642. Thus, all target genes of the 11 tsRNAs were identified and analyzed through intersection analysis, as shown in Figure 6(a) and Supplementary File 5.

According to the following table(Figure 6(b)), 30 target genes were identified through the intersection of target genes of 2 randomly selected tsRNAs from the 11 tsRNAs shown in Figure 6(a). Gene Ontology (GO) pathway analysis was conducted on the 30 genes, as shown in Figure 6(b). Target genes participated in the Alzheimer's disease-presenilin pathway, nicotine pharmacodynamics pathway, Toll receptor signaling pathway, Wnt signaling pathway, and cadherin signaling pathway, among which Alzheimer's disease and Huntington's disease are neurological diseases, further supporting the validation of our results. Two genes (Pcdh9 and Ppp1cb) were revealed from the intersection between the selected signaling pathways and target genes of AS-tDR-002004 or AS-tDR-002583. In other words, Pcdh9, the target gene of AS-tDR-002004, and Ppp1cb, the target gene of AS-tDR-002583, were both involved in the cadherin signaling pathway and the nicotine pharmacodynamics pathway, respectively. Next, we investigated the effects of AS-tDR-002004 and AS-tDR-002583 on the expression levels of Pcdh9 and Ppp1cb. The AS-tDR-002004 mimic inhibited Pcdh9 expression, and the AS-tDR-002583 mimic suppressed Ppp1cb expression (Figure 6(c)). Hence, these results indicated that AS-tDR-002004 or AS-tDR-002583, as targets of XFZYD therapy for TBI, alleviated TBI via the cadherin signaling pathway and the nicotine pharmacodynamics pathway.

### 3.6. Biological Function Analysis Revealed Potential Therapeutic Effects of tsRNAs

**Figure 7:**
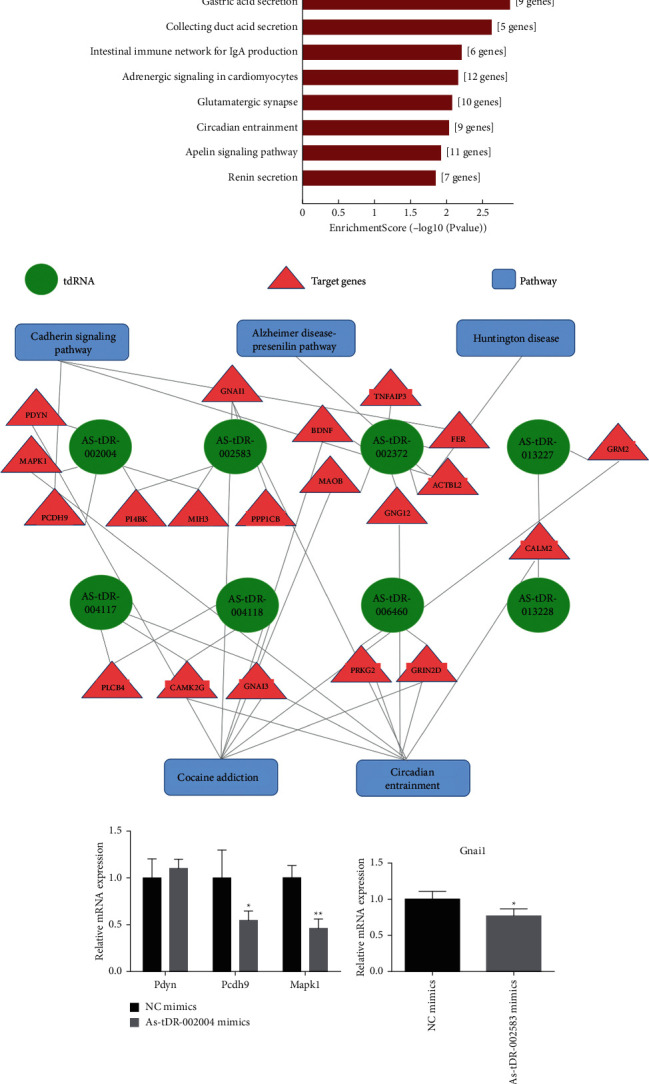
Biological function analysis revealing potential therapeutic effects of tsRNAs. (a) The top 10 enriched terms are shown, ranked by *p* value and colored by the number of enriched genes. (b) TsRNA-mRNA-pathway interaction networks. (c) qPCR detected target gene levels of overexpressed AS-tDR-002004/AS-tDR-002583 in PC12 cells. ^*∗*^*p* < 0.05 and ^*∗∗*^*p* < 0.01 indicate significant differences compared with NC mimics.

tsRNAs can regulate mRNA translational activities, and hence, to understand their biological functions, we conducted a bioinformatics analysis of the functions of the target genes. KEGG pathway analysis was executed to explore the functions of target genes. KEGG enrichment analysis identified pertussis, cocaine addiction, gastric acid secretion, collecting duct acid secretion, intestinal immune network for IgA production, adrenergic signaling in cardiomyocytes, glutamatergic synapse, circadian entrainment, apelin signaling pathway, and renin secretion functions. Moreover, cleavage of growing transcripts in the termination region (conducted; 6 genes enriched) was the main term conducted by Reactome Gene Sets. Ranked by *p* values, the top 10 enriched terms are shown in Figure 7(a) and Supplementary File 6. Among those, cocaine addiction and circadian entrainment were the related pathways of brain function.

There were 13 genes enriched in cocaine addiction and the circadian entrainment signaling pathway (Bdnf, Gnai1, Gnai3, Grin2d, Grm2, Maob, Cam2, Camk2g, Gng12, Mapk1, Pcb4, Prkm2, and Pdyn). We analyzed these 13 genes and the target genes of AS-tDR-002004 or AS-tDR-002583 in Figure 6(b) with the signaling pathways involved in these genes (Figure 7(b)). These genes were regulated by 8 tsRNAs. Mapk1 was the target gene of AS-tDR-002004, and Gnai1 was the target gene of AS-tDR-002583. After overexpressing AS-tDR-002004 mimics in PC12 cells, Pcdh9 and Mapk1 were significantly downregulated (*p* < 0.01, *p* < 0.05), and Pdyn showed no change (all *p* > 0.05) (Figure 7(c)). When transfected with AS-tDR-002583 mimics, GNAI1 was significantly downregulated (*p* < 0.05) (Figure 7(c)).

## 4. Discussion

Traumatic brain injury (TBI) is a growing public health problem worldwide and is a leading cause of death and disability [26]. Although major progress has been made in understanding the pathophysiology of this injury, this has not yet led to substantial improvements in outcome due to a lack of treatments, which have proven successful during phase III trials for modern medicine [27]. XFZYD has been used for years to treat TBI in China and has been demonstrated to be effective in clinical practice [28]. However, the underlying mechanism remains unknown. Previous studies have merely partially deciphered the molecular mechanism of XFZYD in treating TBI. The present study was intended to explore the tsRNA expression profile in a rat model of TBI before and after XFZYD therapy. Briefly, our RNA-Seq data illustrate that 11 tsRNAs were mediated by XFZYD. Our qPCR assay validated that 2 tsRNAs (AS-tDR-002004 and AS-tDR-002583) were potential targets for XFZYD therapy against TBI. Thereafter, we aimed to analyze the corresponding target genes, that is, Pi4kb and Mlh3. Intriguingly, our results identified a reciprocal relationship between the abundance of tsRNAs and these two target genes in the rat model. For further verification, 11 targeted genes of tsRNAs were analyzed, and 30 intersected target genes were identified in our results. Additionally, GO analysis highlighted signaling pathways related to neurological disorders and adhesion, further making our results convincing. Subsequently, Pcdh9 was found to correspond to AS-tDR-002004, while Ppp1cb corresponded to AS-tDR-002583 through the intersection between 30 target genes and the target genes of 2 tsRNAs (AS-tDR-002004 and AS-tDR-002583). Finally, the 2 tsRNAs were overexpressed or inhibited in PC12 cells to determine the resulting expression alterations of Pi4kb, Mlh3, Pcdh9, and Ppp1cb. AS-tDR-002004 and AS-tDR-002583 were confirmed as targets for XFZYD therapy against TBI that could alleviate TBI through the cadherin signaling pathway and the nicotine pharmacodynamics pathway. Furthermore, biological function analysis revealed the potential therapeutic effects of tsRNAs, and the results showed that Mapk1 and Gnai1 were related genes for XFZYD therapy against TBI. Most of the human genome is composed of ncRNAs, which are extensively involved in diverse biological and pathologic activities. tsRNAs are the most common type of small ncRNAs and have been reported to participate in the modulation of RNA processing and protein translation [5, 29]. Mounting evidence has shed light on the close association between tsRNAs and various biological processes as well as human diseases, including tumors, diseases of the cardiovascular system, epigenetics, and neurological disorders [20]. Ramos et al. reported that a remarkable number of neurodevelopmental disorders have been linked to defects in tRNA modifications [30]. Karaiskos et al. also detected tRFs originating from the 3'- and 5'-ends of tRNAs in rat brains at significant levels and illustrated the utility of tRF analysis for annotating tRNA genes in sequenced genomes [31]. However, the mechanism of tsRNAs in TBI remains undefined. Our results further clarify the mechanisms of tsRNAs in TBI.

TBI is a growing public health problem worldwide and is a leading cause of death and disability [32]. Neuroscientists and surgeons tend to search for potential novel drugs from traditional Chinese medicine libraries to treat TBI [33]. The traditional Chinese medicine “XFZYD” possesses the ability to improve blood circulation and disperse stasis, while it has also been elucidated as a reliable and effective therapy for multiple diseases, including unstable angina pectoris, coronary artery diseases, thromboembolic stroke, ischemic stroke, and TBI [5, 12]. It is mainly composed of flavonoids, organic acids, terpenoids, and steroid saponins, among other components. It has been reported that XFZYD is potentially functional in the alleviation of TBI from antidepression and synaptic regulation perspectives, which are concordant with our study [34]. However, research regarding the detailed biological mechanism of XFZYD underlying TBI is in the incipient stage. Our work further authenticated the alleviating effects of XFZYD therapy in rats with TBI and identified the corresponding drug targets, establishing a foundation to reveal the pharmacological mechanism and offering evidence for the clinical application of integrative medical treatment in therapy using traditional Chinese and Western medicines.

## 5. Conclusions

Our work successfully illuminates the efficiency of XFZYD for the treatment of TBI. This study initially revealed the changed expression patterns of tsRNAs in the hippocampi of CCI rats after XFZYD treatment. tsRNAs might be novel potential therapeutic targets by which XFZYD regulates TBI-induced biological pathways. The present study provides a basis and direction for future investigations to explore the mechanisms by which XFZYD protects against long-term neurological deficiencies after TBI. The present work may provide valuable evidence for further clinical application of XFZYD for treating TBI. The interaction process between tsRNAs and mRNAs needs to be clarified clearly by more studies.

## Data Availability

Specific study data are available from the authors upon request.

## Abbreviations

tRNA: tRNA-derived small RNAs
XFZYD: Xuefu-Zhuyu Decoction
TBI: Traumatic brain injury
KEGG: Kyoto Encyclopedia of Genes and Genomes
GO: Gene Ontology
CCI: Controlled cortical impact.

## Supplementary Materials

Supplementary Materials
*Supplementary File 1*. Statistical analysis for properly annotated tRFS. *Supplementary File 2.* The total precisely matched tsRNAs identified from tsRNA-Seq. (A) indicates the sham group; (B) indicates the TBI group; (C) indicates the XFZYD group. *Supplementary File 3*. The significantly dysregulated tsRNAs from tsRNA-Seq. (A) indicates the sham group; (B) indicates the TBI group; (C) indicates the XFZYD group (FC > 1.3 and *p* < 0.05). *Supplementary File 4.*Target genes of AS-tDR-002004 and AS-tDR-002583. *Supplementary File 5.* All target genes of 11 tsRNAs were identified and analyzed. *Supplementary File 6*. Top 10 enriched terms ranked by *p* value.
